# Small-Molecule NANT Therapeutics Targeting the Brain–Immune Axis in Alzheimer’s Disease: Mechanisms, Clinical Progress, and Translational Challenges

**DOI:** 10.3390/molecules31183274

**Published:** 2026-09-16

**Authors:** Niti Sharma, Seong Soo A. An

**Affiliations:** Lab of Aging-Related Diseases, Bionano Research Institute, Gachon University, Seongnam-si 461-701, Republic of Korea; nitisharma@gachon.ac.kr

**Keywords:** Alzheimer’s disease, NANT, small molecules, neuroinflammation, therapeutics

## Abstract

Alzheimer’s disease (AD) is now widely accepted as a complex disorder involving multiple interconnected pathological processes. Increasing evidence suggest that neuroinflammation and immune system dysregulation actively contribute to neurodegeneration, extending beyond the traditional view that the disease is driven solely by amyloid-β (Aβ) and tau pathology. With the advent of studies showing anti-amyloid antibodies to have only modest effects with significant toxicity, there has been a growing interest in investigating non-amyloid non-tau (NANT) treatment approaches. This review provided a general review of small molecules for neuroinflammation-based NANT therapies in AD. We reviewed several agents that affect the brain/immune axis, namely, kinase inhibitors (nelflamaimod and masitinib), NLRP3 inflammasome inhibitors (MCC950, selnoflast, and dapansutrile), TREM2 activators (VG-3927), gingipain inhibitors, PPARγ activators, KCa3.1 inhibitors, and repurposed drugs such as ambroxol and cromolyn. Some common drawbacks were identified for the drugs that failed to provide their anticipated effects. It was found that they all suffered from late-stage treatment, patient heterogeneity, inadequate central nervous system penetration, and poorly designed preclinical studies. Additional translational challenges included sex-specific differences in neuroimmune responses, APOE ε4-associated immune dysfunction, and the need for biomarker-guided patient selection. Although immune-targeted therapies remain strongly supported by biological evidence in AD, meaningful clinical progress will likely require earlier intervention strategies guided by biomarkers and therapies capable of targeting multiple disease pathways simultaneously.

## 1. Introduction

Alzheimer’s disease (AD) continues to be a major global health concern, affecting millions of older adults and creating growing challenges for healthcare systems, caregivers, and society. According to recent statistics, more than 7.4 million Americans aged 65 years and older are living with Alzheimer’s dementia, while the annual cost of care is expected to reach nearly $409 billion [1]. Unfortunately, after decades of extensive research in this field, effective treatments that would be capable of changing the clinical course of AD are still scarce. The initial approaches to AD research were based on the amyloid cascade hypothesis, which suggested that the aggregation of β-amyloid peptides initiated all subsequent stages of the pathophysiological process, including the appearance of pathological alterations in tau proteins, synapses, and neurons [2]. Recent findings indicated that inflammation and immune dysregulation might occur earlier in the brain, possibly at the very first stages of development preceding cognitive impairment and actively participating in AD pathogenesis [3,4]. Current knowledge on the pathology of AD indicates that the disease is formed by several mechanisms acting together. Apart from amyloid-β and tau, a range of other factors like oxidative stress, mitochondrial abnormalities, damage to the blood–brain barrier, and alteration in signaling pathways involving immune system molecules might contribute to the AD progression [5,6,7,8]. The limited efficiency of interventions targeting only amyloid or tau proteins prompted researchers to investigate other aspects of disease etiology and progression mechanisms. Current Food and Drug Administration (FDA)-approved treatments, including acetylcholinesterase inhibitors (donepezil, rivastigmine, galantamine) and the NMDA receptor antagonist memantine, mainly provide symptomatic relief. They do not stop disease progression [9]. More recently, anti-amyloid antibodies such as lecanemab and donanemab showed modest clinical benefits, but they also carry notable side effects [10,11]. Even with these therapies, cognitive decline was only moderately slowed (around 27–35%), suggesting that Aβ is not the sole driver of AD pathology [12,13,14,15,16]. Adding to this complexity, only about 10% of patients exhibit “pure” AD, while the vast majority have mixed pathologies [17]. The disconnect between amyloid removal and meaningful clinical improvement showed AD complexity and suggested that no single-pathway model could fully explain its progression. In addition to AD, Aβ has been implicated in many other diseases such as type 2 diabetes, heart disease, retinal neurodegeneration, traumatic brain injury, and Parkinson’s disease [18,19,20], which again showed the complexity involved in Aβ biology and the inadequacy of target-based therapies.

Due to these shortcomings, neuroinflammation and immune dysregulation has become an area of growing interest within the NANT approach. Activation of microglia and astrocytes, oxidative stress, the destruction of the BBB, and changes in the peripheral immune system all have an impact on the development of neuronal injury in the case of AD [21,22]. Over time, these processes result in a chronic inflammatory process that affects synaptic transmission and reduces neuroplasticity [23]. The importance of immunological components for the development of AD was evidenced by results obtained during genetic studies. There were multiple risk genes associated with the immune system, such as TREM2, cluster of differentiation 33 (CD33), complement receptor 1 (CR1), and APOE [24,25]. Dementia in elderly patients is always accompanied by various types of diseases, such as vascular injury, TDP-43 accumulation, metabolic changes, and chronic inflammation. As a result, treatments targeted exclusively to amyloid and tau pathology are unlikely to provide desired results due to multiple coexisting pathogenic processes [26,27,28]. Due to this complexity, small molecules to target neuroinflammation gained increasing attention as potential NANT therapeutics. These compounds are attractive because of their low molecular weight (<900 Da), oral bioavailability, BBB penetration, and affordability. They also offer the potential to alter multiple interconnected disease pathways concurrently and thus might provide broader neuroprotective effects. Their structures could further be improved to enhance efficacy and reduce toxicity.

In this review, the focus was on small-molecule neuroinflammatory inhibitors within the context of the NANT theory, particularly in terms of the mode of action and the treatment potential of these compounds. In addition, the ability of these compounds to tackle the multifaceted nature of the pathogenesis associated with AD was also explored. The compounds discussed were selected to provide a representative overview of small-molecule strategies targeting different components of the brain–immune axis. Accordingly, both clinically advanced candidates and selected preclinical compounds were included. Attention was also given to translational challenges that may influence therapeutic success, including patient heterogeneity, apolipoprotein E (APOE ε4)-associated immune dysfunction, sex-specific differences in neuroimmune responses, biomarker-guided patient selection, and the potential role of combination therapies. While clinical-stage compounds offer important translational insights, early-stage compounds were incorporated because they target emerging neuroimmune pathways and provide mechanistic evidence that may inform future therapeutic development.

## 2. Methods

This article was prepared as a narrative review. The relevant literature was identified through searches of PubMed, Scopus, Web of Science, and ClinicalTrials.gov up to June 2026. Search terms included combinations of “Alzheimer’s disease”, “neuroinflammation”, “immune dysregulation”, “brain–immune axis”, “microglia”, “inflammasome”, “NLRP3”, “TREM2”, “small molecules”, “non-amyloid non-tau”, “NANT”, “kinase inhibitors”, “PPARγ agonists”, “gingipain inhibitors”, “KCa3.1”, and “Alzheimer’s clinical trials”. Additional articles were identified through citation tracking of relevant publications. Priority was given to peer-reviewed studies, clinical trials, and recent reviews relevant to neuroinflammation-targeted therapeutic strategies in AD.

## 3. The Brain–Immune Axis in AD

The brain was considered an immunologically privileged organ for a long time since it was relatively safe due to its location behind the BBB, which restricted the entry of peripheral immune cells. While immune surveillance was thought to rely primarily on resident cells within the central nervous system, this understanding has changed considerably over the past decade. It is now established that the brain actively communicates with the peripheral immune system via multiple pathways, such as meningeal lymphatic vessels, perivascular spaces, and circumventricular organs [29,30]. Collectively, these anatomical sites have come to be known as the brain–immune axis [31].

In the healthy brain, microglia constantly sense the environment for any signs of distress, while astrocytes help maintain synaptic function and regulate inflammatory signaling [32]. Under normal circumstances, these immune responses are tightly controlled and get resolved once the threat is removed [33]. In AD, however, there is sustained activation of microglia due to long-term exposure to misfolded proteins, neuronal trauma, and metabolic stress, which results in an overproduction of inflammatory cytokines like interleukin-1 beta (IL-1β), interleukin-6 (IL-6), and tumor necrosis factor alpha (TNF-α), causing synaptic dysfunction and neurodegeneration [34]. This inflammatory response is partly regulated by the TREM2 signaling pathway, as its dysfunction impairs these responses, resulting in reduced clearance of toxic proteins and worsening chronic neuroinflammation [35]. As AD is marked by BBB dysfunction, peripheral immune cells such as monocytes, CD8^+^ T cells, and natural killer cells migrate to the brain and exacerbate neuroinflammation [36]. Additionally, the malfunctioning of the meningeal lymphatic system hinders the removal of inflammatory molecules and protein aggregates from the brain, leading to their accumulation [37]. This causes a feedback loop involving chronic inflammation, impaired waste clearance, and progressive neurodegeneration. Additionally, systemic inflammation also impacts neuroinflammation in AD [38]. The peripheral immune cells, which get activated due to the systemic inflammation, attain a more pro-inflammatory state when entering the brain tissue, thus causing even more damage to neurons [39,40]. Thus, further research into brain–immune communication is essential for developing new AD therapies. In AD, neuroinflammation develops through continuous interaction between activated microglia, reactive astrocytes, and infiltrating immune cells. Together, these processes maintain a chronic inflammatory state that eventually harms synapses and neurons [23]. These mechanisms are closely linked, so targeting only amyloid or tau is unlikely to stop disease progression. This shifted the focus toward inflammatory pathways within the NANT approach. Small molecules offered one possible strategy. They could modulate microglial activity, stabilize mast cells, and inhibit kinase signaling. Importantly, they do this without directly targeting amyloid or tau pathology.

The brain–immune axis comprises a complex network of interactions between resident central nervous system cells, peripheral immune components, and systemic regulatory pathways that collectively influence brain homeostasis and disease progression. In AD, dysregulation of this network contributes to chronic neuroinflammation and neurodegeneration. While microglial activation has received considerable attention, growing evidence indicate that additional mechanisms, including complement signaling, gut–brain communication, and adaptive immune responses, also play important roles in shaping the neuroimmune landscape of AD [41,42,43,44].

### 3.1. Complement-Mediated Immune Dysregulation in AD

The complement system has emerged as an important contributor to neuroinflammation and immune dysregulation in AD. Beyond its classical role in innate immunity, complement signaling participates in synaptic remodeling and clearance of cellular debris within the central nervous system [45]. However, excessive or chronic activation of complement pathways may promote pathological synapse elimination and neurodegeneration. Genetic studies identified complement receptor 1 (CR1) as a risk factor for late-onset AD, supporting complement dysregulation in disease susceptibility [46]. In addition, clusterin (CLU, apolipoprotein J), another major AD risk gene, regulates complement activation and may influence amyloid-β aggregation and clearance [47,48]. Increasing evidence suggested that aberrant complement activation contributed to microglial-mediated synaptic loss and sustained neuroinflammatory responses, highlighting the complement cascade as an important component of the brain–immune axis in AD [45,49].

### 3.2. Gut–Brain Axis and Neuroinflammation

Growing evidence indicate that alterations in the gut microbiota could influence AD pathogenesis through bidirectional communication between the gastrointestinal tract and the central nervous system [50,51]. Gut dysbiosis promotes systemic inflammation, alters the production of microbial metabolites, and affects immune signaling pathways that regulate microglial function. Increased intestinal permeability further facilitates the translocation of inflammatory mediators into the circulation, thereby amplifying neuroinflammatory responses [52,53]. Associations between microbial imbalance and cognitive decline have been observed, suggesting a potential role of the gut–brain axis in AD progression [54,55,56,57]. Although the precise mechanisms remained under investigation, modulation of the gut microbiome emerged as a promising strategy for regulating neuroinflammation and restoring immune homeostasis in AD [58].

### 3.3. Adaptive Immunity and CD8^+^ T-Cell Infiltration

Although AD has traditionally been viewed as a disorder driven primarily by innate immune mechanisms, increasing evidence has supported the involvement of adaptive immune responses [59,60]. Infiltrating CD8^+^ T cells were identified within the brains of patients with AD, particularly in regions associated with neurodegeneration [61,62]. These cells may have contributed to disease progression through the release of pro-inflammatory cytokines and cytotoxic mediators that exacerbate neuronal injury [63]. Furthermore, interactions between infiltrating T cells, microglia, and other immune cells may have amplified neuroinflammatory signaling within the brain [64,65]. The recognition of adaptive immune involvement expanded the concept of the brain–immune axis beyond microglial activation alone and suggested that both innate and adaptive immune pathways contributed to AD pathogenesis [66].

Collectively, these findings emphasized the multifaceted nature of immune dysregulation in AD. Therefore, effective therapeutic strategies might need to target multiple interconnected pathways rather than a single inflammatory mechanism.

## 4. Neuroinflammation-Targeting Small Molecules

**Figure 1:**
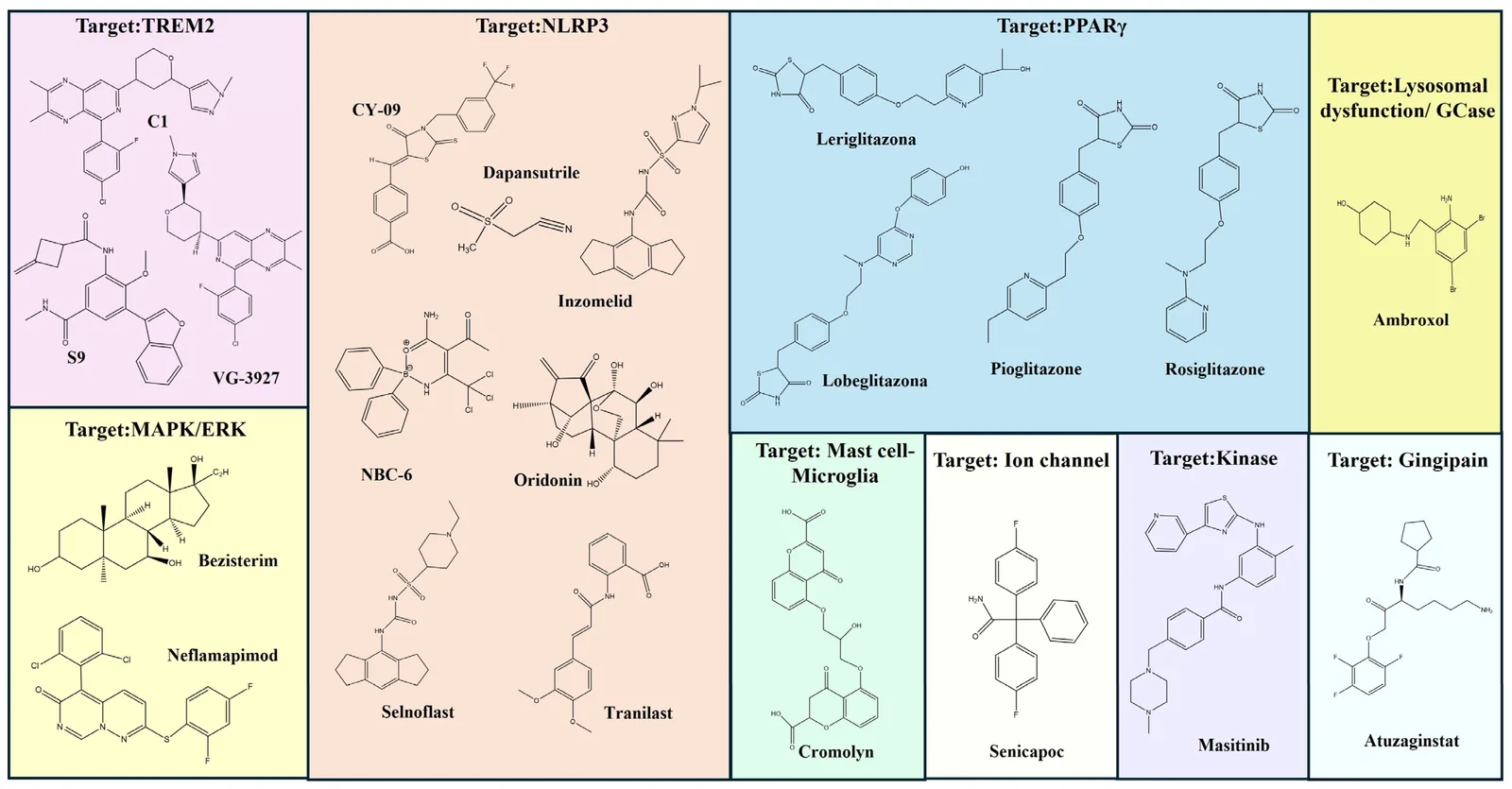
Some important small molecules targeting neuroinflammation. [Chemical structures were prepared using ChemDraw Pro 12.0 based on compound structures obtained from the PubChem database].

Early attention focused on the potential role of non-steroidal anti-inflammatory drugs (NSAIDs) in AD prevention. Long-term use of NSAIDs was repeatedly linked to decreased AD risk, suggesting a link between cyclooxygenase (COX)-dependent neuroinflammation and neurodegeneration [67,68]. Mechanistically, this association is attributed to the role of COX enzymes in regulating prostaglandin-mediated neuroinflammation [69]. COX-2 is induced in neurons, astrocytes, and activated microglia in response to Aβ and inflammatory stimuli, resulting in increased prostaglandin production that promotes microglial activation, inflammatory cytokine release, and neuronal injury. These inflammatory mediators also impair microglial Aβ clearance, thereby contributing to disease progression [70]. However, because conventional NSAIDs non-selectively inhibited both COX isoforms, they broadly suppressed prostaglandin signaling rather than selectively targeting the detrimental inflammatory pathways [71]. Consequently, the promising epidemiological findings were not translated into clinical benefit. The AD Anti-inflammatory Prevention Trial (ADAPT), together with clinical trials of ibuprofen, rofecoxib, and indomethacin, failed to demonstrate meaningful cognitive benefit in symptomatic patients [72,73]. This lack of efficacy suggested that broad COX inhibition alone was insufficient to address the complex neuroimmune mechanisms underlying AD and highlighted the need for more selective NANT approaches targeting the brain–immune axis [73]. Some of the important small molecules targeting neuroinflammation are discussed below (Figure 1).

### 4.1. Kinase Inhibitors

Kinase inhibitors are one of the most advanced small-molecule drugs targeting neuroinflammation in AD. They disrupt intracellular signaling pathways that lead to microglial activation and cytokine secretion. Neflamapimod (VX-745) selectively inhibits p38α mitogen-activated protein kinase (p38α MAPK) with a 9 nM IC_50_ (50% inhibitory concentration) value. p38α MAPK is the crucial enzyme in the inflammatory signaling pathways of microglia and neurons [74,75]. Excessive activation of p38α MAPK in AD increases TNF-α and other pro-inflammatory cytokines, contributing to neuroinflammation, synaptic dysfunction, and impaired endosomal recycling and long-term potentiation [76]. By p38α MAPK inhibition, neflamapimod promoted microglial homeostasis and synaptic preservation in preclinical and randomized clinical evaluations [77]. In a completed Phase 2b study in patients with Lewy body dementia (LBD), a disease characterized by neuroinflammation similar to that of AD, neflamapimod led to robust cognitive and neurodegeneration biomarker benefits [78], supporting the validity of p38α MAPK as a drug target until further clinical testing in AD.

In contrast, masitinib adopts a broader anti-inflammatory approach by inhibiting several tyrosine kinases, including the receptor tyrosine kinases c-Kit (CD117) and platelet-derived growth factor receptor (PDGFR), as well as the Src-family non-receptor tyrosine kinase Lyn, in both microglia and mast cells [79,80]. c-Kit regulates mast cell development, survival, and activation, whereas Lyn mediates downstream signaling that triggers mast cell degranulation and the release of pro-inflammatory mediators. By simultaneously inhibiting c-Kit and Lyn, masitinib suppresses both mast cell activation and the subsequent inflammatory cascade, providing broader immunomodulatory effects than targeting a single downstream mediator [79]. Inflammation in the brain is triggered by mast cells present in the meninges and perivascular areas, which releases factors leading to activation of microglia and destruction of the BBB [81]. Thus, by stabilizing both mast cells and microglia simultaneously, masitinib offers a dual-acting anti-neuroinflammatory profile. Masitinib broadly addresses the neuroinflammatory relevance of tyrosine kinase inhibition across neurodegenerative conditions like multiple sclerosis (MS) [82] and amyotrophic lateral sclerosis (ALS) [83].

Masitinib has been evaluated as an add-on to standard therapies, including cholinesterase inhibitors and/or memantine, in patients with mild-to-moderate AD. In a Phase 3 clinical trial, significant improvements in cognition and daily functioning, assessed using the Alzheimer’s Disease Assessment Scale-Cognitive Subscale (ADAS-cog), were observed in a pre-specified subgroup of patients, whereas the primary endpoint was not met in the overall intention-to-treat population [84,85]. Subsequently, the U.S. Food and Drug Administration (FDA) Peripheral and Central Nervous System Drugs Advisory Committee voted against approval in 2023 because of concerns regarding the statistical analysis and subgroup selection. Yet, AB Science continued to evaluate masitinib as an add-on therapy in patients with mild AD [86]. Although masitinib demonstrated encouraging findings in selected patients, its clinical efficacy requires further confirmation in adequately designed studies [85].

### 4.2. NLRP3 Inflammasome Inhibitors

The NLRP3 inflammasome is one of the more discussed inflammasome pathways in AD research [87]. Under physiological conditions, it helps immune cells respond to stress and cellular damage. In AD, this balance is disturbed. Aβ accumulation, mitochondrial dysfunction, and lysosomal stress all seem to push the system into a persistent activated state. Once activated, NLRP3 promotes the release of pro-inflammatory cytokines such as IL-1β and IL-18, which aggravates neuronal injury [87]. Importantly, abnormal NLRP3 activation was also detected in peripheral immune cells such as monocytes and macrophages, indicating that inflammation outside the brain may further influence neuroinflammatory processes in AD [40,88].

These findings heightened interest in NLRP3 as a therapeutic target. Among the available inhibitors, MCC950 was the first compound to show strong preclinical efficacy in streptozotocin (STZ)-treated SHSY-5Y cells and rat models with AD [89]. It selectively blocks NLRP3 activation by binding to the Walker B motif of the NLRP3-NACHT domain. This blocks ATP hydrolysis and prevents inflammasome assembly [90]. In transgenic AD mice, MCC950 reduced amyloid burden, enhanced microglial phagocytosis, restored autophagy, and improved cognitive performance [89,91,92,93]. Despite these promising findings, MCC950 did not advance into clinical trials for AD. Safety concerns, particularly dose-dependent hepatotoxicity observed in preclinical trials, represented a major limitation [94]. Even so, the compound became a reference point for the field. New inhibitors were designed using its scaffold and binding behavior as a template. CY-09 is one example. It showed selectivity, BBB penetration, and anti-inflammatory effects in preclinical systems [95,96,97]. Another NLRP3 inhibitor, emlenoflast (inzomelid; IZD174) had reached early clinical evaluation. A Phase 1 single ascending dose/multiple ascending dose (SAD/MAD) trial involving 80 healthy volunteers and an open-label preliminary study in patients with cryopyrin-associated periodic syndromes (CAPS) reported acceptable safety and tolerability [98]. Dose-dependent target engagement was also suggested, although full results were not published.

Selnoflast is currently one of the most advanced purpose-designed NLRP3 inhibitors in clinical development. A Phase 1b trial of selnoflast in early PD enrolled 60 participants across 17 centers in Europe and the United States and evaluated 28 days of treatment versus placebo. The drug was generally well tolerated, with headache and nausea being the most common adverse events, and no serious adverse events were reported. Importantly, selnoflast demonstrated target engagement by reducing IL-1β production in peripheral blood cells and IL-18 levels in the cerebrospinal fluid (CSF) by approximately 30%. Although exploratory clinical outcomes showed slight improvement in treated patients compared with the placebo, the differences were not statistically significant [99]. Roche is currently evaluating selnoflast for reducing vascular inflammation associated with atherosclerosis [100].

OLT1177 (dapansutrile), a β-sulfonyl nitrile compound, inhibits ATPase activity, thereby preventing NLRP3 assembly and reducing production of downstream pro-inflammatory cytokines [101]. In APP/PS1 AD mouse models, it reduced hippocampal IL-1β, attenuated microglial activation, and improved spatial memory through a mechanism independent of amyloid or tau [102]. It also improved motor functions and reduced α-synuclein toxicity and inflammation in synucleinopathy with nigral neurodegeneration PD models [103]. OLT1177 has completed Phase 2 trials in gout and heart failure, establishing a human safety record [104]. Currently, it is being evaluated as an anti-inflammatory therapy in PD [105].

Tranilast (TR), originally developed as an antiallergic drug, was found to directly inhibit NLRP3 by blocking its oligomerization through interaction with the NACHT domain [106,107]. Its established clinical safety profile makes it an attractive repurposed candidate for neuroinflammatory disorders. TR reduced NLRP3-dependent IL-1β release and exerted anti-inflammatory effects in preclinical experiments [106,108,109,110]. TR can modulate adaptive immune responses by suppressing B- and T-cell proliferation, inducing T-cell cycle arrest, and regulating the T helper cells (Th1/Th2) balance [108]. It also reduced the release of inflammatory mediators such as interferon gamma (IFN-γ), IL-2α, and chemokine (CXC motif) ligand 9/10 (CXCL9/10) and suppressed pathogenic T-cell responses in autoimmune disease models, including rheumatoid arthritis (RA) and MS [111,112,113]. Still, despite modest CNS penetration, oral bioavailability, and a suitable safety profile [107], tranilast did not enter AD clinical trials, and its relatively lower potency compared with newer NLRP3 inhibitors suggested that further optimization may be needed to achieve sufficient brain target engagement.

Oridonin (ori), a natural diterpenoid from *Rabdosia rubescens*, covalently binds cysteine 279 on NLRP3, blocks NEK7 recruitment and prevents apoptosis-associated speck-like protein containing a CARD (ASC) adaptor assembly [114]. Ori was found to exert multiple neuroprotective activities on an Aβ_1–42_-induced mouse model of AD. It reduced Aβ_1–42_-induced synaptic loss both in vivo and in vitro. Ori also enhanced the formation of dendrites and spines in hippocampal neurons in the same AD model. In addition, Ori promoted the expression of synaptic proteins and activated the brain-derived neurotrophic factor/tyrosine receptor kinase b/cyclic AMP (cAMP) response element-binding (BDNF/TrkB/CREB) signaling pathway, important for synaptic plasticity and memory. The molecular effects observed included enhanced cognitive functions [115]. In 5× FAD transgenic mice models, ori attenuated the production of pro-inflammatory cytokines in microglia by inhibition of the receptor-interacting protein kinase 1-extracellular signal-regulated kinase 1/2-nuclear factor kappa B (RIPK1-ERK1/2-NF-κB) signaling pathway, which plays a key role in regulating inflammation and cell survival [116]. Furthermore, ori improved spatial learning and memory, reduced Aβ plaque deposition, and attenuated inflammatory and necroptotic markers through suppression of RIPK1-RIPK3-MLKL-mediated signaling in 5× FAD mice [116]. Moreover, ori also reduced neuronal loss in ischemic stroke models by inhibition of RIPK3-mediated excessive mitophagy, further supporting its neuroprotective potential [117]. Even though there was strong evidence of neuroprotective activity, no clinical trial of Ori was registered for any neurological disorder. The clinical development of oridonin was limited by its poor bioavailability and unfavorable pharmacokinetic properties, highlighting the need for improved delivery systems.

JC-171 is a structure-based small-molecule NLRP3 inhibitor that targets the leucine-rich repeat domain of NLRP3, giving it a distinct selectivity profile compared with many other inhibitors in this class [118]. JC-171 delayed disease progression and reduced severity in experimental autoimmune encephalomyelitis (EAE), a mouse model of MS, in both preventive and therapeutic settings. These effects were associated with reduced IL-1β production and suppression of pathogenic Th17 immune responses [118].

NBC6 is an oxanorbornene molecule developed as a selective NLRP3 inhibitor identified through high-throughput screening [119]. In cellular neuroinflammation models, NBC6 reduced ASC speck formation, caspase-1 activation, and IL-1β maturation [119,120]. Although mechanistically promising, in vivo evidence in neurodegenerative models remain limited.

### 4.3. TREM2 Agonists

Interest in TREM2 grew after rare TREM2 variants were linked to a higher risk of late-onset AD, highlighting the important role of immune-cell dysfunction in disease pathogenesis [121,122,123]. TREM2 is mainly expressed by microglia in the brain and modulates cell survival, phagocytosis, metabolism, and the transformation of cells into beneficial disease-associated microglia responsible for amyloid removal and synapse support [124,125]. Defective TREM2 signaling leads to inefficient amyloid removal, abnormal inflammatory modulation, poor cell survival, and inadequate protective mechanisms near amyloid plaques [124]. Consequently, the brain’s immune response becomes increasingly inefficient, leading to chronic neuroinflammation and neurodegeneration.

Small-molecule TREM2 agonists aimed to restore microglial phagocytosis, survival, and anti-inflammatory signaling through pathways such as phosphoinositide 3-kinase/protein kinase B (PI3K/AKT) and DNAX activation protein 12/spleen tyrosine kinase (DAP12/SYK) [126,127]. These compounds were able to overcome some limitations associated with antibody-based therapies, particularly poor brain penetration, and were associated with improved microglial signaling, enhanced phagocytosis, and synaptic protection in cellular studies [126,128]. VG-3927 is currently the most clinically advanced small-molecule TREM2 agonist, having recently completed a Phase 1 clinical trial with positive results that demonstrated acceptable safety, tolerability, and preliminary target engagement [129,130]. This compound is advancing into Phase 2 development [130]. Preclinical compounds such as C1 and S9 showed the ability to activate TREM2 signaling and enhanced microglial phagocytosis through different mechanisms, supporting their potential for further development [126,128,131].

Other than VG-3927, C1, and S9, research on the small-molecule TREM2 agonist is still at an early stage. Researchers are increasingly applying structure-based virtual screening and AI-assisted drug discovery to identify compounds targeting the TREM2 ligand-binding domain, but only a limited number of molecules have been characterized so far in AD-relevant models. In addition, the field still lacks reliable pharmacodynamic biomarkers to confirm whether TREM2 agonists are producing sufficient microglial activation in the human brain, making dose optimization and clinical trial design particularly challenging [132].

### 4.4. Gingipain Inhibitors

An emerging hypothesis on AD suggests that chronic oral infection with *Porphyromonas gingivalis* might contribute to neuroinflammation and neurodegeneration through bacterial proteases known as gingipains. Gingipains were detected at higher levels in postmortem AD brains and were shown to damage neuronal proteins, activate complement pathways, stimulate microglial inflammation, and impair the clearance of neurotoxic proteins [133,134]. Thus, chronic peripheral infection could lead to sustained activation of the immune system and neuroinflammation via BBB disruption and microglial priming, making gingipain inhibition an effective NANT therapy [135].

Atuzaginstat (COR388) was developed as a selective small-molecule inhibitor of *P. gingivalis* gingipain proteases and showed promising preclinical effects, including reduced neuroinflammation, microglial activation, and bacterial burden [136]. However, the Phase 2/3 GAIN trial failed to meet its primary cognitive endpoints and showed hepatotoxicity at higher doses [137]. A second-generation compound, COR588 (later developed clinically as LHP588), was subsequently developed with improved metabolic stability and reduced hepatic exposure and is currently recruiting for Phase 2 evaluation in AD [138] after successful Phase 1 results [139].

### 4.5. PPARγ Agonists

Peroxisome proliferator-activated receptor gamma (PPARγ) is a nuclear receptor involved in both metabolic regulation and immune signaling, making it an important target in AD [140,141]. In microglia and peripheral macrophages, PPARγ activation promotes an anti-inflammatory state by reducing NF-κB-driven cytokine production, enhancing phagocytic clearance, and supporting BBB integrity [141]. PPARγ is also linked to brain insulin signaling, which is frequently impaired in AD and contributes to neuroinflammation, synaptic dysfunction, and reduced neuronal energy metabolism [142,143].

Pioglitazone and rosiglitazone, two thiazolidinedione-class PPARγ agonists, were originally approved for type 2 diabetes (T2D). They were later investigated for AD treatment because of their anti-inflammatory and insulin-sensitizing properties. Reduced neuroinflammation, improved mitochondrial function, and neuroprotective effects were seen in preclinical models [144,145]. However, these benefits were not clearly reproduced in clinical studies, which showed little or no significant improvement in cognition or overall function in patients with AD [146,147]. One major limitation was poor effective brain exposure. Pioglitazone was actively transported out of the brain by P-glycoprotein (P-gp) efflux transporters at the BBB, whereas rosiglitazone showed even lower brain penetration [148].

Leriglitazone (MIN-102) was developed to address the poor brain penetration observed with earlier PPARγ agonists [149]. Its chemical structure was modified to reduce P-gp-mediated efflux, enabling improved BBB penetration in human adrenoleukodystrophy (ALD) and Friedreich’s ataxia [149,150,151]. In the preclinical setup, leriglitazone reduced microglial activation, decreased pro-inflammatory cytokine release, and improved mitochondrial function through PPARγ-dependent pathways [152]. Leriglitazone-mediated PPARγ activation might support axonal myelination, protect neurons and astrocytes, and help preserve BBB integrity [153,154,155]. Although no AD-specific clinical trial was reported, leriglitazone remains the only PPARγ agonist with confirmed human brain penetration, making it a promising candidate for future neurodegenerative disease studies [154].

Lobeglitazone is a thiazolidinedione approved for T2D in South Korea [156] and had shown a cardioprotective effect in T2D patients with ischemic stroke [157]. Lobeglitazone also has a favorable long-term safety profile, effective glucose-lowering activity, and durable glycemic control in real-world clinical settings [158,159]. It had shown potential neuroprotective effects in intracerebral hemorrhage (ICH) models by reducing brain edema and neuroinflammation through inhibition of the IL-1β-ERK-COX-2 signaling pathway [160]. In addition, lobeglitazone partially improved motor defficits and reduced inflammatory markers such as TNF-α and NF-κB in a diabetic rat model of PD [161], further supporting its anti-inflammatory and neuroprotective potential.

However, no neurological clinical trials have evaluated lobeglitazone in humans.

### 4.6. KCa3.1 Channel Inhibitors

Senicapoc is an extremely potent small-molecule inhibitor of the calcium-activated potassium channel known as KCa3.1 (or Gardos channel and KCNN4) [162]. KCa3.1 is expressed on microglia, T cells, mast cells, and peripheral macrophages and plays an important role in immune-cell migration and inflammatory signaling [163]. Mechanistically, KCa3.1-mediated K^+^ efflux maintains the membrane potential required for sustained Ca^2+^ influx during immune-cell activation [164]. The resulting increase in intracellular Ca^2+^ activates downstream inflammatory signaling pathways that promote the production of reactive oxygen species, nitric oxide, and pro-inflammatory cytokines in microglia [165]. Therefore, pharmacological inhibition of KCa3.1 interrupts this calcium-dependent inflammatory cascade while largely preserving normal immune-cell viability and function [163]. In ischemic stroke models, senicapoc reduced infarct size, improved neurological recovery, and suppressed microglial activation, T-cell infiltration, and inflammatory mediators, including inducible nitric oxide synthase (iNOS), COX-2, and NLRP3 [166,167]. The compound also demonstrated sufficient brain target engagement and did not interfere with tissue plasminogen activator (tPA) activity, supporting its potential as an adjunctive neuroprotective therapy [167]. Across multiple clinical studies involving hundreds of patients across the United States, senicapoc was found to be orally available, metabolically stable, safe, and well tolerated [162]. In 5x FAD mice models, senicapoc reduced microglial activation, neuroinflammation, and cerebral amyloid burden while improving hippocampal synaptic plasticity. KCa3.1 activity was also increased in postmortem AD brains and 5x FAD mice, further supporting this channel as a potential therapeutic target in AD [168]. At present, the drug is in a Phase 2 clinical trial in patients with mild or prodromal AD [169].

### 4.7. Emerging and Repurposed Neuroimmune Modulators

#### 4.7.1. MAPK/ERK Signaling Modulators

Bezisterim (NE3107) is a novel agent with a unique structure that functions as a metabolic and anti-inflammatory modulator without producing any of the side effects caused by full PPARγ agonists, such as fluid retention and fat production. It mainly serves as an ERK signaling inhibitor, resulting in a reduction in the production of pro-inflammatory mediators like TNF-α and IL-6 and improved insulin signaling [170]. Mechanistically, chronic ERK activation impairs insulin receptor substrate-1 (IRS-1) signaling, contributing to both neuronal insulin resistance and neuroinflammation [171]. By inhibiting ERK, NE3107 restores insulin signaling while reducing NF-κB-mediated inflammatory cytokine production, thereby targeting two interconnected pathological processes implicated in AD [172]. In a Phase 2 clinical study for AD, NE3107 exhibited promising results [170,173], and its combined metabolic and anti-inflammatory actions have also generated interest in the treatment of other neurodegenerative disorders, including PD [174].

#### 4.7.2. Lysosomal Dysfunction/GCase Enhancement

Ambroxol (ABX), widely used in respiratory medicine, has recently gained attention in neurodegeneration research because of its ability to enhance glucocerebrosidase (GCase) activity [175]. This is particularly relevant in AD, where impaired lysosomal function and reduced GCase activity in microglia can promote NLRP3 inflammasome activation, defective autophagy, and chronic neuroinflammation [176,177,178]. By improving lysosomal homeostasis, ABX reduces upstream drivers of microglial dysfunction and toxic protein accumulation [179,180,181]. ABX enhances GCase activity and promotes autophagy-lysosomal clearance of α-synuclein, supporting its potential disease-modifying effects [182]. Based on these findings, the ABX in new and early Dementia with Lewy Bodies (ANeED) trial was launched to evaluate the safety, tolerability, and potential therapeutic benefits in patients with DLB [183]. In the AiM-PD Phase 2 trial, ABX showed good CNS penetration, increased CSF GCase activity, and was well tolerated during long-term treatment. [184,185]. At present, it is gearing up for its Phase 3 trial [186].

#### 4.7.3. Microglial Modulation and Aβ Clearance

Another repurposing strategy focused on targeting peripheral immune signaling and mast cell-mediated neuroinflammation. Cromolyn sodium was originally developed as an antiallergic drug for asthma. It was later investigated for AD treatment because of its ability to modulate peripheral immune responses and neuroinflammation [187,188]. In preclinical models, cromolyn reduced Aβ42 burden by promoting non-inflammatory microglial clearance of Aβ. It also lowered soluble Aβ40 and Aβ42 levels and improved neuroprotection in transgenic AD mice [189,190,191]. The compound reduced the release of inflammatory cytokines and chemokines, including IL-1β, IL-6, IL-8, IFN-γ, CCL2, and CXCL10, in activated human microglial cells [187]. Modified derivatives such as fluorinated cromolyn were also developed to improve BBB penetration while retaining anti-inflammatory activity [192]. Based on these findings, cromolyn entered a randomized 1/2 Phase study of the ALZT-OP1 combination therapy in AD [193]. It demonstrated good safety and tolerability but failed to meet its primary cognitive endpoint in the overall study population.

#### 4.7.4. p38α MAPK Inhibition

MW150 (MW01-18-150SRM) is a brain-penetrant and highly selective p38α mitogen-activated protein kinase (MAPK) inhibitor developed from the earlier lead compound MW01-2-069A-SRM [194]. Unlike amyloid- or tau-directed therapies, this drug discovery program targeted p38α MAPK, a key regulator of glial activation and pro-inflammatory cytokine production. In preclinical studies, MW01-2-069A-SRM suppressed Aβ-induced upregulation of IL-1β and TNF-α in the hippocampus and attenuated associated synaptic dysfunction and behavioral deficits [195]. The optimized compound, MW150, reduced glial release of pro-inflammatory cytokines and prevented or reversed cognitive deficits in APP/PS1 and APP knock-in mouse models without altering amyloid plaque burden [196], supporting a mechanism independent of direct amyloid targeting. Following favorable safety and tolerability findings in Phase 1 studies, MW150 advanced to Phase 2a clinical evaluation in patients with mild-to-moderate AD [197].

A summary of the mechanisms, developmental progress, and neuroinflammatory relevance of these small molecules is presented in Table 1.

**Figure 2:**
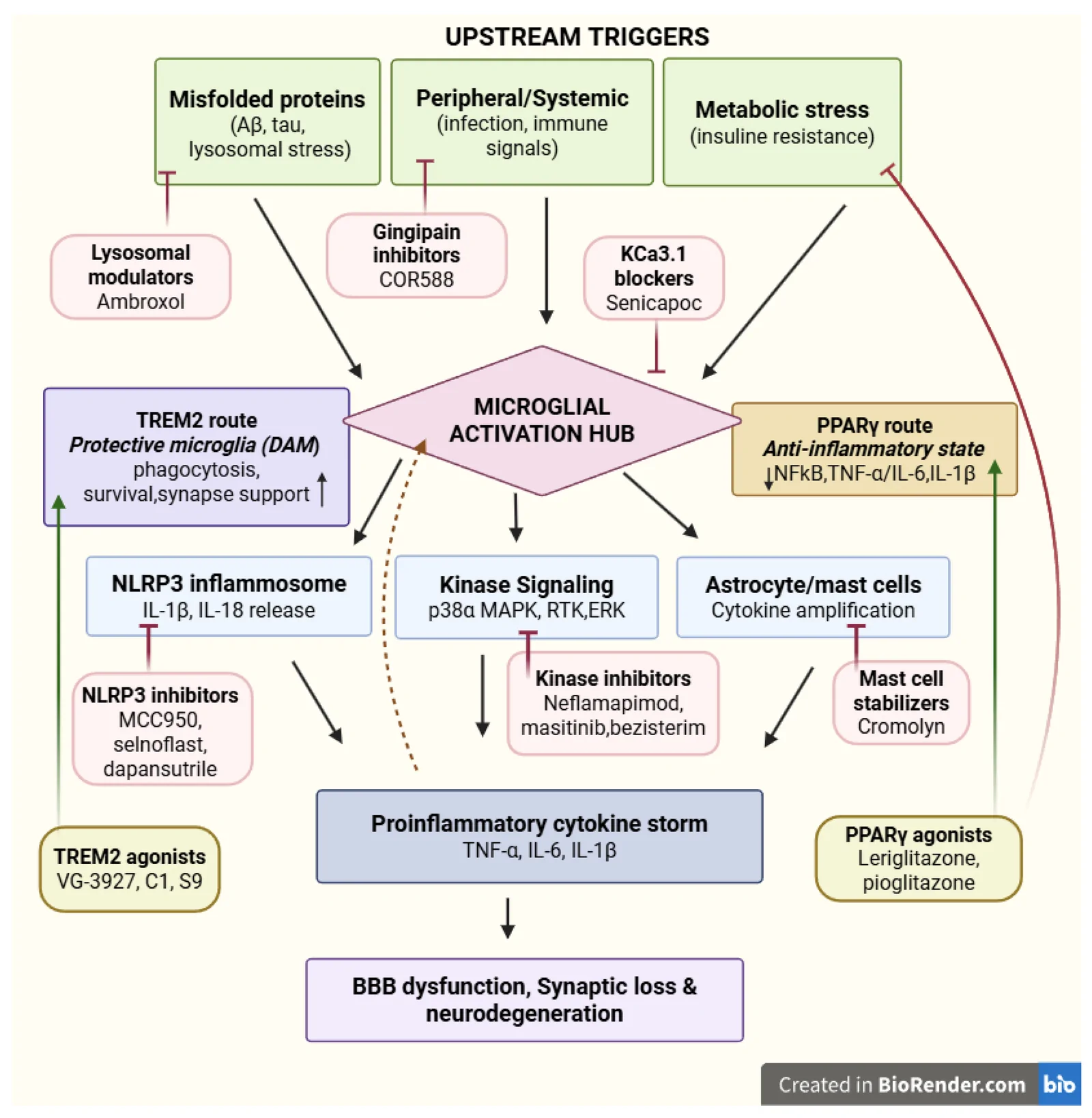
Neuroinflammatory cascade in Alzheimer’s disease and the non-amyloid, non-tau (NANT) small-molecule classes targeting the brain–immune axis. Upstream triggers: misfolded protein accumulation (Aβ, tau) and lysosomal stress, peripheral and systemic immune signals including *Porphyromonas gingivalis* infection, and metabolic stress arising from insulin resistance converge on chronic microglial activation, the central hub of the cascade. Activated microglia drive three interconnected effector arms: assembly of the NLRP3 inflammasome with release of IL-1β and IL-18; pro-inflammatory kinase signaling (p38α MAPK, receptor tyrosine kinases, and ERK); and astrocyte and mast-cell amplification. These converge on a sustained pro-inflammatory cytokine milieu (TNF-α, IL-6, IL-1β) and blood–brain barrier breakdown, culminating in synaptic loss, neurodegeneration, and cognitive decline. A self-perpetuating feedback loop (dashed arrow) returns from the downstream inflammatory output to microglial activation, sustaining chronic neuroinflammation and underlying the limited efficacy of late, single-target intervention. Small-molecule classes are positioned at their primary site of action: gingipain inhibitors and lysosomal modulators act on upstream triggers; TREM2 agonists and KCa3.1 channel blockers act at the microglial hub; NLRP3 inhibitors, kinase inhibitors, and PPARγ agonists act on the effector arms, with PPARγ agonists additionally relieving metabolic stress. Collectively, these interconnected processes represent key manifestations of immune dysregulation in AD. Arrowheads denote activating or restorative actions (TREM2 and PPARγ agonists); flat-ended connectors (⊣) denote inhibitory or blocking actions, the green arrows indicate activation. Abbreviations: Aβ, amyloid-β; BBB, blood–brain barrier; ERK, extracellular signal-regulated kinase; IL, interleukin; KCa3.1, intermediate-conductance calcium-activated potassium channel; MAPK, mitogen-activated protein kinase; NLRP3, NOD-, LRR- and pyrin domain-containing protein 3; PPARγ, peroxisome proliferator-activated receptor gamma; TNF-α, tumor necrosis factor-alpha; TREM2, triggering receptor expressed on myeloid cells 2. Figure created in Biorender.com.

An overview of the neuroinflammatory cascade and the NANT small-molecule classes acting at each node is provided in Figure 2.

## 5. Translational Challenges and Future Directions

Despite major advances in understanding the neuroimmune mechanisms underlying AD, the clinical translation of neuroinflammation-targeting small molecules remains inconsistent. Importantly, however, the many failures in this field do not necessarily invalidate the biological targets themselves but instead feature broader challenges related to the timing of therapeutic intervention, patient heterogeneity, CNS exposure, and translational model design. Additional considerations, including sex differences, APOE ε4 status, biomarker-guided patient selection, and the need for combination therapies, can further influence the successful translation of these approaches into clinical practice.

### 5.1. Timing of Therapeutic Intervention

Neuroinflammatory alterations begin many years before the onset of clinical symptoms, and by the time patients are enrolled in clinical trials, substantial synaptic and neuronal loss may already be irreversible. This issue was illustrated by early NSAID research, in which long-term NSAID use was associated with a lower risk of AD in epidemiological studies, whereas clinical trials in symptomatic patients failed to demonstrate meaningful therapeutic benefit [204,205]. This discrepancy was largely explained by a timing paradox (prevention vs. treatment) and reflects the fact that long-term NSAID users often begin treatment earlier in life, before AD-related pathological processes have fully developed [206]. Moreover, as the COX expression changes in the different stages of AD pathology [207], NSAIDs may benefit only during the earliest phases of AD, when initial Aβ deposition and microglial activation begins but lose efficacy once chronic neuroinflammation and amyloid pathology become established. At later stages, prolonged inhibition of activated microglia may even interfere with Aβ clearance mechanisms [208]. In addition, early observational findings were likely influenced by methodological biases, including healthier baseline characteristics in NSAID users and confounding related to disease stage-dependent prescribing patterns [206,209]. Similar limitations may have also contributed to the disappointing outcomes observed with pioglitazone and rosiglitazone [145]. The COR388 trial failed to meet its primary cognitive and functional endpoints and was additionally associated with hepatotoxicity [136]. Current evidence suggests that therapies targeting neuroinflammation may be more effective when introduced in the early or preclinical stages of AD, before significant neuronal loss occurs. This has increased interest in preventive and early-intervention strategies for high-risk groups, including cognitively normal APOE ε4 carriers.

### 5.2. Translational Limitations of Preclinical Models

One persistent challenge in AD research is the frequent failure of promising preclinical findings translating into successful clinical outcomes. Commonly used animal models, especially transgenic mice, reproduced only limited components of the disease and could not fully mimic the complexity of human immune and glial responses in the brain [210]. Because of these shortcomings, researchers started using alternative systems that might better reflect human biology. Models based on human induced pluripotent stem cells (iPSCs), including microglial cultures and brain organoids, are increasingly explored as tools for studying disease mechanisms and evaluating potential therapies [211,212]. Still, animal models remain the main platform for preclinical testing.

### 5.3. Patient Heterogeneity and the Need for Multi-Pathway Targeting

The inflammatory response observed in AD is highly complex and involves several overlapping biological processes rather than a single abnormal pathway. Activation of microglia, dysfunction of astrocytes, altered peripheral immune signaling, vascular injury, and metabolic abnormalities all contribute to disease progression and influence one another [4]. Since these mechanisms are interconnected, interventions aimed at only one pathway may have limited therapeutic impact. For this reason, future treatment strategies will likely need to target multiple components of the neuroimmune system simultaneously to achieve more meaningful clinical outcomes.

### 5.4. Sex Differences in Neuroinflammation and Treatment Response

Sex influences both neuroinflammatory responses and therapeutic outcomes in AD. Women account for approximately two-thirds of AD cases and exhibit differences in microglial activation, inflammatory signaling, and disease progression compared with men [213,214,215]. Hormonal changes, particularly the decline in estrogen during menopause, may further affect immune regulation in the brain [213,216]. Despite these observations, most NANT clinical trials had not reported sex-specific outcomes. Future studies are expected to incorporate sex-stratified analyses to determine whether treatment efficacy, safety, or dosing differ between men and women.

### 5.5. APOE ε4 Genotype and Innate Immune Dysfunction

The APOE ε4 allele is the strongest genetic risk factor for late-onset AD and plays an important role in neuroimmune regulation [217]. APOE ε4 is associated with impaired microglial function, reduced amyloid clearance, and increased inflammatory signaling [218,219]. As a result, APOE ε4 carriers may respond differently to neuroinflammation-targeting therapies than non-carriers [217]. Incorporating the APOE genotype into clinical trial design may improve patient stratification and help identify individuals most likely to benefit from specific NANT-based interventions.

### 5.6. Biomarker Strategies for Patient Selection and Trial Design

Biomarkers have become increasingly important for the development of neuroinflammation-targeting therapies. Fluid biomarkers such as GFAP, YKL-40, sTREM2, and inflammatory cytokines could provide insights into astrocyte and microglial activity, while imaging biomarkers might help assess disease burden and treatment response [220,221]. Biomarker-guided patient selection may improve trial efficiency by identifying individuals with prominent neuroinflammatory pathology. In addition, biomarkers could provide evidence of target engagement and support the evaluation of therapeutic efficacy.

### 5.7. Rationale for Combination Therapy

As AD is driven by multiple interconnected pathological processes, interventions targeting a single pathway often produce limited therapeutic benefits. Combination approaches involving neuroinflammation-targeting agents together with amyloid-, tau-, metabolic-, or vascular-directed therapies may offer a more comprehensive strategy by addressing multiple disease mechanisms simultaneously. Several combination approaches have already entered clinical evaluation. For example, the ALZT-OP1 program combined cromolyn with low-dose ibuprofen to target both Aβ pathology and neuroinflammation, while the DIAN-TU platform evaluated the combination of the anti-amyloid antibody lecanemab with the anti-tau antibody E2814, reflecting increasing interest in multi-target therapeutic strategies. These studies highlight the growing recognition that modifying multiple disease pathways simultaneously may provide greater therapeutic benefit than targeting a single mechanism alone. Such approaches are increasingly recognized as a promising direction for AD treatment, although challenges related to safety, trial design, and patient selection remain to be resolved.

### 5.8. Shift Toward Precision Neuroimmunology in Clinical Translation

Clinical translation remains another bottleneck. Many therapies that showed promise in preclinical studies failed to replicate their effects in patients. This stressed the need for better trial design. Earlier intervention, careful biomarker-based patient selection, and direct evidence of target engagement in the brain may improve future outcomes. The clinical implementation of combination therapies also presents several practical challenges. Combining neuroinflammation-targeting agents with anti-amyloid or anti-tau therapies may increase the risk of additive adverse effects and drug–drug interactions while complicating the interpretation of treatment efficacy. Furthermore, combination trials generally require larger sample sizes and more complex study designs to distinguish the contribution of each therapeutic component. Biomarker-guided patient selection and evidence of target engagement will therefore be essential for identifying patients most likely to benefit from specific therapeutic combinations and for optimizing future precision medicine approaches in AD.

Despite these challenges, continued advances in biomarker discovery, patient stratification, and the understanding of brain–immune interactions provide a strong foundation for the future development of neuroinflammation-targeting therapies. Together, these advances support a shift toward precision neuroimmunology, in which treatments can be tailored to individual disease mechanisms and combined with complementary therapeutic approaches to improve clinical outcomes in AD.

## 6. Conclusions

Growing evidence indicate that neuroinflammation is central to AD, broadening therapeutic strategies beyond the traditional focus on amyloid and tau. The small molecules described in this review interact with various aspects of the neuroinflammatory process, including microglia activity, inflammasome pathways, impaired lysosomes, interactions with the peripheral immune system, metabolism disorders, and BBB impairment. Importantly, it should be noted that such therapies were intended to restore the balance in the neuroimmune system rather than suppress inflammation. Although no small molecule demonstrated definitive disease-modifying effects in AD through neuroinflammation targeting, major advances were made in neuroimmunology and biomarker discovery. Significant progress was achieved in CNS-directed drug development and precision-based clinical trial design. Future progress in this field will likely depend on the integration of precision medicine approaches. Consideration of factors such as APOE ε4 genotype, sex-specific differences in neuroimmune responses, and biomarker-guided patient stratification may improve the identification of individuals most likely to benefit from neuroinflammation-targeting therapies. In addition, combination strategies that address multiple pathological pathways simultaneously may offer greater therapeutic potential than single-target approaches. These advances support further exploration of neuroinflammatory modulation in AD. Eventually, small molecules targeting neuroinflammation might serve as an important addition to amyloid- and tau-based therapies within a more comprehensive treatment approach for AD. As knowledge of brain–immune interactions continue to expand, these therapies hold promise for advancing AD care beyond symptomatic relief toward disease-modifying outcomes.

## Figures and Tables

**Table 1:** Small-molecule therapeutics in neuroinflammation: mechanisms of action and clinical development.

Target	Compound /Drug	Molecular Formula and Weight (g/mol)	Development Stage	Key Relevance in AD	Ref
NLRP3 Inflammosome	CY-09	C_19_H_12_F_3_NO_3_S_2_ 423.4	Preclinical	Restored cerebral glucose metabolism and reduced inflammasome activation in AD	[95,96,97]
	Dapansutrile (OLT1177)	C_4_H_7_NO _2_S 133.1	Phase 2 trial (DAPA-PD) in PD (NCT07157735) Recruiting	Reduced IL-1β production and neuroinflammation in AD mouse models	[102,103,105]
	Inzomelid (Emlenoflast; IZD174)	C_19_H_24_N_4_O_3_S 388.5	Phase 1 (NCT04015076) completed in healthy adults, demonstrating favorable tolerability and linear pharmacokinetics	Block caspase 1, stop production of pro-inflammatory cytokines	[98]
	JC-171	C_16_H_17_ClN_2_O_5_S 384.8	Preclinical	Reduced neuroinflammation and cognitive deficits in AD mouse models	[118]
	NBC6 *	C_18_H_16_BCl_3_N_2_O_2_ 409.5	Preclinical	Inhibited ASC speck formation and IL-1β maturation	[119,120]
	Oridonin	C_20_H_28_O_6_ 364.4	Preclinical	Suppressed the secretion of pro-inflammatory cytokines, blocked the activation of the RIPK1-RIPK3-MLKL signaling, improved spatial learning and memory performance, decreased Aβ plaque deposition, and attenuated inflammatory and necroptotic markers in both cortical and hippocampal regions	[114,115,116,117]
	Selnoflast (RO7486967)	C_20_H_29_N_3_O_3_S 391.5	Clinical trial: To evaluate the efficacy, safety, pharmacodynamics and pharmacokinetics in reducing vascular inflammation in participants with atherosclerosis at risk for major adverse cardiac events (RIVULET; NCT07448038) Recruiting	Reduced peripheral IL-1β and CSF IL-18; one of the most advanced purpose-designed NLRP3 inhibitors	[100]
	Tranilast	C_18_H_17_NO_5_ 327.3	Preclinical	Suppresses inflammasome activation and modulates T-cell inflammatory responses	[106,107,109,110,112]
TREM2 Signaling	C1	C_24_H_23_ClFN_5_O 451.9	Preclinical	Enhanced microglial phagocytosis and synaptic protection	[4,128]
	S9 *	C_23_H_23_N_2_O_4_ 391.1	Preclinical	Increased phagocytic gene expression and microglial activation	[126]
	VG-3927 ((2R,4S) stereoisomer of C1)	C_24_H_23_ClFN_5_O 451.9	Phase 1 (NCT06343636) completed Showed favorable safety, CNS penetration, and dose-dependent target engagement Based on these findings, a once-daily 25 mg dose is planned for Phase 2 evaluation in AD	Improved microglial activation, and showed preliminary target engagement	[128,129,130,131]
PPARγ Agonist	Leriglitazone (MIN-102)	C_19_H_20_N_2_O_4_S 372.4	Phase 2/3 (NCT05819866) To evaluate efficacy and safety in adult male subjects with cerebral adrenoleukodystrophy Recruiting	Reduced microglial activation and improved mitochondrial function	[149,150,151,153,154,198]
	Lobeglitazone	C_24_H_24_N_4_O_5_S 480.5	Preclinical in neurodegeneration Safety evaluation in T2D clinical trials (NCT02480465)	Reduced IL-1β/ERK/COX-2 signaling and neuroinflammation	[156,157,158,159,160,161]
	Pioglitazone	C_19_H_20_N_2_O_3_S 356.4	Repurposed/Phase 3 (TOMMORROW; NCT01931566) failed Ineffective in delaying AD symptoms	Anti-inflammatory, preserves synapses, reduced tau phosphorylation	[147]
	Rosiglitazone	C_18_H_19_N_3_O_3_S 357.4	Repurposed/Phase 3 (NCT00550420 and NCT00490568) failed No significant improvement in cognition or overall global function	Anti-inflammatory, preserves synapses, reduced tau phosphorylation	[146]
MAPK/ERK Signaling	Bezisterim (NE3107)	C_21_H_30_O_3_ 330.5	Phase 2 (NCT05227820) completed in AD Positive trends in improving cognitive and functional performance Phase 2 (SUNRISE-PD trial; NCT06757010) Result expected in mid-2026	Reduced inflammatory biomarkers and improved metabolic signaling	[199,200]
	Neflamapimod (VX-745)	C_19_H_9_Cl_2_F_2_N_3_OS 436.3	Phase 2b (RewinD-LB trial; NCT05869669) completed Significant improvements in cognitive and neurodegenerative biomarkers in DLB patients	Improved synaptic function and reduced inflammatory signaling	[78]
Lysosomal Dysfunction/GCase Enhancement	Ambroxol	C_13_H_18_Br_2_N_2_O 378.1	Phase 2 (NCT02914366) completed and advancing to Phase 3 for PD (ASPro-PD trial; NCT05778617)	Improved lysosomal function and reduced neuroinflammatory signaling	[175,179,180,181,183,184,185,186]
Gingipain Inhibitors	Atuzaginstat (COR388)	C_19_H_25_F_3_N_2_O_3_ 386.4	Phase 2/3 (GAIN trial; NCT03823404) completed Failed to show significant overall cognitive benefits and faced an FDA clinical hold due to liver toxicity concerns	Targeted *P. gingivalis*-associated neuroinflammation	[137]
	COR588;LHP588 *	NA	Phase 1 (NCT04920903) completed single- and multiple-ascending dose study evaluating the safety, tolerability, and pharmacokinetics Phase 2 (SPRING trial; NCT06847321) AD Recruiting	Designed to improve safety and pharmacokinetics over COR388	[138,201,202]
Mast Cell–Microglia Modulation	Cromolyn	C_23_H_16_O_11_ 468.4	Phase 3 combination ALZT-OP1 trial (COGNITE; NCT02547818) completed ALZT-OP1 showed acceptable safety and adequate CSF drug penetration in Phase 1/2 (NCT04570644), while the Phase 3 trial enrolled 620 early AD patients to evaluate the efficacy and safety of cromolyn–ibuprofen combination therapy using CDR-Sum of Boxes as the primary endpoint	Reduced inflammatory cytokines and promoted non-inflammatory Aβ clearance	[187,190,192,193]
Multi-target Kinase Inhibition	Masitinib	C_28_H_30_N_6_OS 498.6	Phase 2b/3 study (NCT01872598) and Phase 3 (NCT05564169) completed for AD Significant improvements in both cognition (ADAS-cog) and daily functioning Phase 2b/3 (NCT02588677); Phase 3 (NCT03127267) for ALS Recruiting	Reduced mast cell and microglial inflammatory signaling	[83,203]
Ion Channel Modulation	Senicapoc	C_20_H_15_F_2_NO 323.3	Phase 2 (NCT04804241) proof-of-mechanism study Recruiting	Reduced microglial activation and inflammasome-associated signaling	[166]
p38α MAP Kinase Inhibitor	MW150	C_24_H_23_N_5_ 381.4	Phase 2a, mild-to-moderate AD (NCT05194163)	Inhibits p38 MAPK, and modulates BDNF/TrkB	[197]

Abbreviations. AD: Alzheimer’s disease; ALS: Amyotrophic lateral sclerosis; BDNF: Brain-derived neurotrophic factor; COX-2: Cyclooxygenase-2; ERK: Extracellular signal-regulated kinase; IL-1β:Interleukin-1 beta; MAPK: Mitogen-activated protein kinase; MLKL: Mixed lineage kinase domain-like protein; NCT: National clinical trial; NEK7: NIMA-related kinase 7; NLRP3: NLR family pyrin domain containing 3; PD: Parkinson’s disease; PDGFR: Platelet-derived growth factor receptor; PPARγ: Peroxisome proliferator-activated receptor gamma; RIPK1: Receptor-interacting serine/threonine-protein kinase 1; RIPK3: Receptor-interacting serine/threonine-protein kinase 3; TREM2: Triggering receptor expressed on myeloid cells 2; TrkB: Tropomyosin receptor kinase B. Footnote: Molecular formulas and molecular weights were verified using PubChem whenever available. * Not indexed in PubChem; molecular information obtained from the original publication (S9: [126])/secondary sources (NBC6:MedChemExpress). COR588 not available (NA).

## Data Availability

No new data were created or analyzed in this study. Data sharing is not applicable to this article.
